# Qualitative and quantitative gastric ultrasound assessment in highly skilled regional anesthesiologists

**DOI:** 10.1186/s12871-021-01550-z

**Published:** 2022-01-03

**Authors:** Rattanaporn Tankul, Pathom Halilamien, Suwimon Tangwiwat, Sukanya Dejarkom, Pawinee Pangthipampai

**Affiliations:** Department of Anesthesiology, Faculty of Medicine Siriraj Hospital, Mahidol University, 2 Wanglang Road, Bangkoknoi, Bangkok, 10700 Thailand

**Keywords:** Qualitative ultrasound assessment, Gastric content, Novice anesthesiologist gastric sonographers, Quantification, Gastric volume, Training

## Abstract

**Background:**

Pulmonary aspiration is a major complication in anesthesia, and various studies have shown that gastric sonography can reliably provide valuable information relative to both the qualitative and quantitative aspects of gastric content. This study aimed to determine the accuracy of ultrasound assessment of gastric content compared between two novice anesthesiologist gastric sonographers.

**Methods:**

This prospective cohort study of two anesthesiologists learning to perform qualitative and quantitative ultrasound assessment of gastric content on healthy volunteers was conducted at Siriraj Hospital (Bangkok, Thailand). This trial was registered with ClinicalTrials.gov (reg. no. NCT04760106).

**Results:**

Of the 50 enrolled participants, three were excluded due to study protocol violation. Each anesthesiologist performed a qualitative assessment on 47 participants for an overall total of 94 scans. There were 15 males and 32 females (age 42 ± 11.7 years, weight 61.2 ± 13.1 kg, height 160.7 ± 7.3 cm, and BMI 23.6 ± 4.3 kg/m^2^). The overall success rate for all gastric content categories was approximately 96%. From antral cross-sectional area measurement, as the ingested volume increased, there was a tendency toward increased deviation from the actual ingested volume. Interrater agreement between anesthesiologists was analyzed using intraclass correlation coefficients (ICCs). A larger fluid volume was found to be associated with a lower level of agreement between the two anesthesiologists. The ICCs were 0.706 (95% CI: −0.125 to 0.931), 0.669 (95% CI: −0.254 to 0.920), 0.362 (95% CI: −0.498 to 0.807) for the 100 ml, 200 ml, and 300 ml fluid volumes, respectively. The mean duration to perform an ultrasound examination for each gastric content category and for the entire examination did not differ significantly between anesthesiologists (*p* > 0.05).

**Conclusion:**

Our results indicate that qualitative ultrasound assessment of gastric content is highly accurate and can be easily learned. In contrast, quantification of gastric volume by novice gastric sonographers is more complex and requires more training.

**Trial registration:**

ClinicalTrials.gov no. NCT04760106 Date registered on Feb 11, 2021. Prospectively registered.

## Background

Pulmonary aspiration is a major complication in anesthesia. Its incidence was reported to be 1 in every 2000 to 3000 elective surgical cases [1]. The prevalence of pulmonary aspiration was reported to be higher in parturients, emergency patients, and in patients with higher American Society of Anesthesiologists (ASA) status [2, 3]. Pulmonary aspiration is associated with significant morbidity and mortality [4–6], so prevention is a key component of anesthesia practice as reflected most prominently in preoperative fasting guidelines [7, 8]. However, these fasting guidelines may not be applicable across all patient populations, especially patients with delayed gastric emptying time from inherent medical and surgical conditions, and in patients with pain [9, 10]. Prandial status can also be further complicated in certain settings, such as dementia patients and emergency surgery [11].

Recent advancement in gastric sonography has enabled anesthesiologists to perform a bedside ultrasound assessment of gastric content and volume. Various studies have shown that gastric sonography can reliably provide valuable information relative to both qualitative (nature of content) and quantitative (volume) aspects of gastric content [9, 12–16]. When implemented with a structured protocol, point-of-care gastric ultrasound can help to determine the risk of pulmonary aspiration, which can facilitate the clinical decision to cancel or proceed with a procedure [17].

Qualitative assessment of gastric ultrasound is considered to be relatively easy to learn. A recent study suggested that a minimum of 33 scans is needed to achieve a 95% success rate in the qualitative assessment of gastric ultrasound [18]. However, since the risk and severity of aspiration are also influenced by gastric volume [19, 20], further study concerning the quantitative assessment of gastric volume is warranted. Kruisselbrink, *et al*. reported the intra- and interrater reliability of ultrasound assessment of gastric volume to be highly satisfactory when performed by an experienced gastric sonographer [14]. However, no study has investigated the level of complexity of quantitative assessment by inexperienced gastric sonographers. Whether or not the same results can be extrapolated to novice gastric sonographers is an area ripe for further investigation.

Despite point-of-care ultrasound (PoCUS) being widely practiced by emergency and critical care physicians, the tool is only slowly gaining recognition among anesthesiologists [21]. In view of its promising application, this study was conducted as an initiative to encourage the use of PoCUS, specifically gastric ultrasound, in the author’s department. Since regional anesthesia is regarded as a subspecialty with a recognized high level of skill in ultrasonography [22], two highly skilled regional anesthesiologists were selected to participate in this study. Moreover, regional anesthesiologists often find themselves in a setting where they have to manage patients without a secure airway [23]. Gastric ultrasound could be a valuable addition to the cache of clinical skills among regional anesthesiologists. Accordingly, this study is conducted to investigate the performance of regional anesthesiologists in the qualitative and quantitative assessment of gastric content.

## Methods

This prospective cohort study of two anesthesiologists learning to perform qualitative and quantitative ultrasound assessment of gastric content on healthy volunteers was conducted at the Department of Anesthesiology of the Faculty of Medicine Siriraj Hospital, Mahidol University, Bangkok, Thailand. This trial was registered with ClinicalTrials.gov (reg. no. NCT04760106). The protocol for this study was approved by the Siriraj Institutional Review Board (SIRB) (COA no. Si 830/2018), and written informed consent to participate was obtained from both anesthesiologists and all healthy volunteers that were recruited for gastric content assessment by ultrasound. The study report has been prepared in accordance with the Transparent Reporting of Evaluations with Nonrandomized Designs (TREND) guidelines.

The two enrolled anesthesiologists are staff anesthesiologists accredited in regional anesthesia. Neither anesthesiologist received any prior training in gastric sonography. This study was conducted over a three-day course, as confined by the availability of the foreign expert sonographer who agreed to kindly assist in this study. Fifty healthy volunteers were recruited as subjects in which gastric assessment would be evaluated. The inclusion criteria were age from 18 to 70 years with an American Society of Anesthesiologists (ASA) physical status of class I to II. Participants having one or more of the following were excluded: body mass index (BMI) >40 kg/m^2^, pregnancy, diabetes mellitus, history of upper gastrointestinal tract disease (including hiatal hernia or gastric tumor), and/or previous surgical procedure on the esophagus, stomach, or upper abdomen. Termination criteria include any volunteer whose ultrasound assessments could not be completed by both anesthesiologists. All ultrasound examinations were performed with a low-frequency (2 to 5 MHz) curved array transducer using a Sonimage HS1 ultrasound system.

### Teaching intervention

The training program included self-directed learning, conventional didactic lectures, and hands-on practice. Online materials, including educational videos and picture library, were accessible for self-study via www.gastricultrasound.org and www.usra.ca. A 1-h lecture was provided by an expert sonographer (>500 gastric scans), followed by a 1-h interactive hands-on workshop on all prandial status (fasting/empty, clear fluid, solid content). Both anesthesiologists were instructed in how to perform a quantitative assessment of gastric content volume using the free-tracing method. Both anesthesiologists were also required to independently perform 10 exams for practice. The performance of the gastric scan complied with the standardized scanning protocol described in a previously published study [14]. The four important principles of the scanning protocol were emphasized, including: 1) imaging the cross-section of the antrum in the sagittal plane at the level of the aorta, 2) positioning the patient in the right lateral decubitus position, 3) measuring gastric content between peristaltic contractions, and 4) measuring the antrum between two sides of the serosa. This scanning protocol was found to be highly reproducible and demonstrated high intra- and interrater reliability.

### Outcome measurement

Participants were informed by telephone to fast for a minimum of 8 h prior to the procedure. On the study date, participants were randomized into one of the five following stomach state categories: empty, fluid (apple juice 100, 200, or 300 ml), or solid (pork with sticky rice). Apple juice was chosen over plain water due to its longer stomach emptying time. The group allocation process was performed by a research assistant, and both anesthesiologists were blinded to each volunteer’s allocation assignment. All ultrasound examinations were performed 5 min after ingestion, except for those allocated to the empty/fasting group.

The primary objective was to determine the accuracy of the qualitative ultrasound assessment of the gastric content performed by regional anesthesiologists. The secondary objectives were to perform a cumulative sum (CUSUM) control chart analysis to evaluate the learning curve of the two included novice anesthesiologist gastric sonographers relative to their ability to qualitatively assess gastric content via ultrasound, determine the interrater reliability of the quantitative ultrasound assessment of gastric volume, and determine the amount of time needed to perform ultrasound examination of each type of gastric content.

For the qualitative assessment, each participant was scanned once by both examiners. The timer started once the ultrasound probe touched the patient until the declaration of answer to the research assistant. Each scan session lasted no longer than 5 min to mitigate the confounding effect of gastric emptying time. The examiners independently performed gastric ultrasound in a random sequence. The results were documented in a case record form. Competence was defined as attaining a 90% success rate [18, 24, 25].

For the quantitative assessment, study volunteers were placed in the right lateral decubitus position and the antral cross-sectional area (CSA) was measured in the sagittal plane. Measurement was performed between peristaltic contractions to prevent underestimation of the gastric content [14]. To minimize the effect of gastric emptying time, the images of the antral CSA were labeled and saved for calculation of gastric volume at the end of the session. The two anesthesiologists performed the quantitative ultrasound independently, and the measurement was compared against the known ingested volume. The formula used for calculation of gastric volume was based on an equation validated in another study [13].

$$\mathrm{Volume}=27.0+14.6\ast \mathrm{Right}-\mathrm{lat}\ \mathrm{CSA}-1.28\ast \mathrm{age}$$

### Statistical analysis

The sample size calculation was based on a previous study by Arzola, *et al*. that reported the accuracy of qualitative assessment of gastric content to be approximately 80% [18]. With an error of 0.12 (15% of 80), a sample size of 43 participants was calculated. To compensate for missing data or participant withdrawal for any reason, fifty participants were recruited and equally randomized into the 5 following groups: empty, 100 ml fluid, 200 ml fluid, 300 ml fluid, and solid.

For the qualitative analysis, the proportion of correct diagnosis for all three content categories (empty, liquid, and fluid) was reported as number and percentage. The duration of time taken to perform an ultrasound scan for the different types of gastric content was reported as mean plus/minus standard deviation, and compared using a generalized estimating equation (GEE). The difference in overall duration taken by the two anesthesiologists was compared using a paired *t*-test.

Cumulative sum (CUSUM) control chart analysis was used to plot the learning curve of both anesthesiologists. CUSUM has been used in various studies to determine the training required to achieve clinical competency [26–29], and for quality control [30]. To perform a CUSUM analysis, four parameters have to be determined, including an acceptable failure rate (*p*_*0*_), an unacceptable failure rate (*p*_*1*_), type 1 (α) error, and type 2 (ß) error. Previous study [18] set the acceptable and unacceptable failure rates at 0.10 and 0.30, respectively, and the type I and type II errors were both set at 0.1. Three variables were then calculated, including *h0*, *h1,* and *s*. The variables *h0* and *h1* denote boundary lines that were used to draw multiples of *h* on the vertical axis. The x-axis represents the number of attempts by the anesthesiologists. Starting at zero, the CUSUM graph ascends by *1-s* for each failed attempt, and descends by *s* for each successful attempt. The anesthesiologists were considered to have achieved competency when the CUSUM graph descends across two boundary lines. In contrast, competency was not achieved if the graph ascends across two boundary lines. The observation is continued if the CUSUM graph remains between *h0* and *h1*.

Regarding quantitative measurement of gastric volume, the calculated volume for each participant by each anesthesiologist was compared against the known ingested volume, and the result was reported as mean plus/minus standard deviation. The calculated volume between the two anesthesiologists was similarly reported. Intraclass correlation coefficient (ICC) was used to evaluate interrater reliability between the two anesthesiologists.

Participant demographic and anthropometric data were summarized using descriptive statistics. Those data are presented as number and percentage for categorical data, and as mean plus/minus standard deviation (normally distributed data) or median and range (non-normally distributed data) for continuous data. SPSS Statistics software (SPSS, Inc., Chicago, IL, USA) was used to perform all statistical analyses, and a *p*-value of <0.05 was considered to be statistically significant.

## Results

**Fig. 1:**
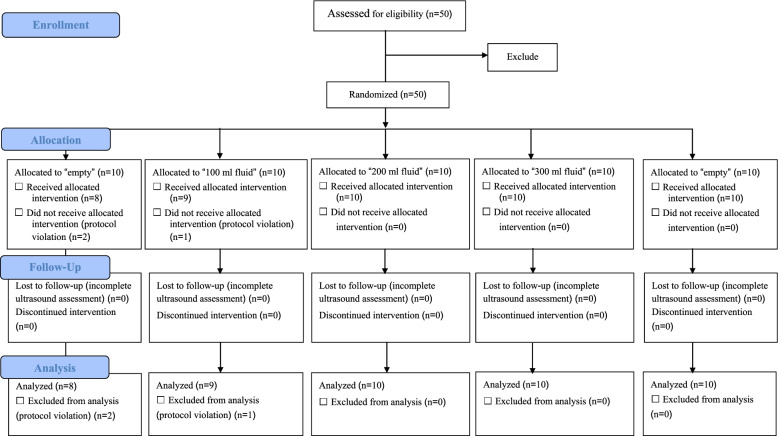
CONSORT flow diagram

Of the 50 enrolled participants, three were excluded due to violation of their designated study protocol. One participant in the empty group ate porridge, and another ate bread before the examination. One participant in the fluid group drank a carbonated beverage prior to the examination. A CONSORT flow diagram describing the study protocol is shown in Fig. 1. The study population comprised 15 males (31.9%) and 32 females (68.1%). Other demographic data are presented in mean plus/minus standard deviation, as follows: age 42 ± 11.7 years, weight 61.2 ± 13.1 kg, height 160.7 ± 7.3 cm, and BMI 23.6 ± 4.3 kg/m^2^.

### Accuracy

Each anesthesiologist performed a qualitative assessment on 47 participants for an overall total of 94 scans. Regarding the nature of the gastric content, both anesthesiologists were able to make a correct diagnosis in almost all participants. In the ‘fluid’ group, both anesthesiologists achieved a 100% success rate. The first anesthesiologist made an incorrect diagnosis in only 1 out of 10 participants in the ‘solid’ group (90% success rate), while the second anesthesiologist made an incorrect diagnosis in 1 out of 9 participants in the ‘empty’ group (88% success rate). (Table 1) The overall success rate for all gastric content categories was approximately 96%.

**Table 1:** Percentage of correct diagnosis for anesthesiologists 1 and 2 compared among the 3 gastric content categories

Anesthesiologists	Gastric content		
	Empty (n = 8), n (%)	Clear fluid (n = 29), n (%)	Solid (n = 10), n (%)
Anesthesiologist 1	8 (100%)	29 (100%)	9 (90%)
Anesthesiologist 2	7 (88%)	29 (100%)	10 (100%)
Both	15 (94%)	58 (100%)	19 (95%)

### Learning curves for qualitative analysis

**Fig. 2:**
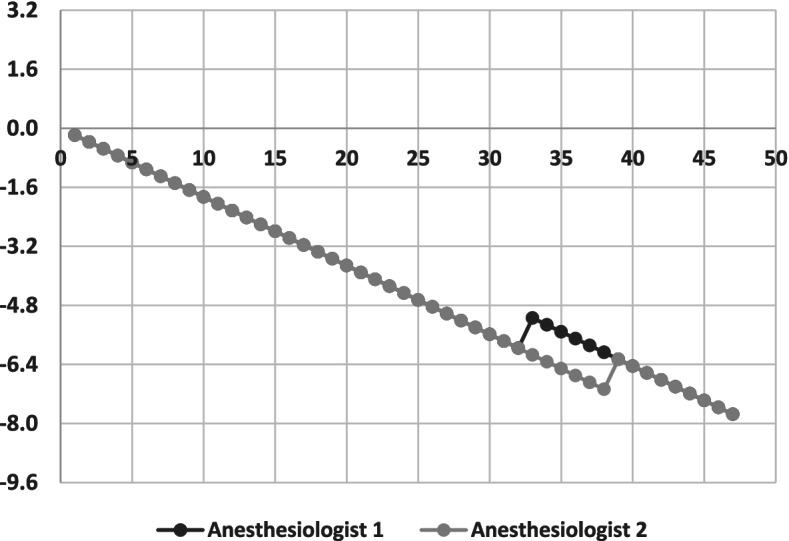
Cumulative sum (CUSUM) control chart graph of the learning curves of the two anesthesiologists. Each point represents a consecutive gastric scan. For each scan, the graph descends by *s* for each successful attempt, and ascends by *1-s* for each failed attempt

The CUSUM control chart graph evaluated the learning curve of both anesthesiologists. Both anesthesiologists successfully achieved competency for the qualitative assessment, which was defined as a 90% success rate or higher within 9 attempts. The CUSUM graph is presented in Fig. 2.

### Quantitative analysis and interrater agreement

Since both anesthesiologists were able to make a correct diagnosis for every participant in the ‘fluid’ group, both went on to perform the antral CSA measurement. The calculated volume by each anesthesiologist is shown in Table 2. The difference between the calculated volume and the known ingested volume is also reported. Of note – as the ingested volume increased, there was a tendency toward increased deviation from the predetermined volume.

**Table 2:** Measured volume for different fluid volumes and mean difference compared against the known ingested volume

Actual fluid volume (ml)	Measured volume (mean ± SD)		Mean difference in volume (±SD)		
	Anesthesiologist 1	Anesthesiologist 2	Actual ***vs.*** Anesthesiologist 1	Actual ***vs.*** Anesthesiologist 2	Anesthesiologist 1 ***vs.*** Anesthesiologist 2
100	126.6 ± 37.8	97.3 ± 46.5	−26.6	2.7	29.3 ± 35.2
200	177.0 ± 43.0	136.7 ± 44.3	23.0	63.3	40.3 ± 33.7
300	200.4 ± 79.6	143.7 ± 18.4	99.6	156.3	56.7 ± 67.8

*Abbreviation*: *SD* standard deviation

Interrater agreement between the two anesthesiologists was analyzed using intraclass correlation coefficients (ICC). As shown in Table 3, a larger fluid volume was found to be associated with a lower level of agreement between the two anesthesiologists. The ICCs were 0.706 (95% CI: −0.125 to 0.931), 0.669 (95% CI: −0.254 to 0.920), 0.362 (95% CI: −0.498 to 0.807) for the 100 ml, 200 ml, and 300 ml fluid volumes, respectively. These findings indicated poor (ICC: <0.5) to moderate (ICC: 0.5–0.75) interrater agreement between the two evaluated anesthesiologists [31].

**Table 3:** Interrater agreement for quantitative gastric ultrasound assessment for different fluid volumes

Actual fluid volume (ml)	Agreement in volume between students	
	No. of volunteers	ICC (95% CI)^**a**^
100	9	0.706 (−0.125 to 0.931)
200	10	0.669 (−0.254 to 0.920)
300	10	0.362 (−0.498 to 0.807)

*Abbreviations*: *ICC* interclass correlation coefficient, *CI* confidence interval

^a^ Two-way random, average measures, absolute agreement

### Duration of the ultrasound examination

The mean duration taken to perform an ultrasound examination for each gastric content category did not differ significantly between the two anesthesiologists (*p* > 0.05). There was also no significant difference between anesthesiologists for the mean overall duration needed to perform a complete ultrasound examination (*p* > 0.05). (Table 4).

**Table 4:** Ultrasound examination duration (in minutes) for anesthesiologists 1 and 2 compared among the 3 gastric content categories; and mean overall duration of ultrasound examination by each anesthesiologist

Anesthesiologists	n	Gastric content categories			
		Empty (n = 8)	Clear fluid (n = 29)	Solid (n = 10)	Overall (n = 47)
Anesthesiologist 1	47	3.8 ± 1.0	3.0 ± 1.1	3.5 ± 1.2	3.25 ± 1.11^A^
Anesthesiologist 2	47	3.0 ± 1.1	3.3 ± 1.1	3.0 ± 1.3	3.17 ± 1.14^A^
Both		3.4 ± 1.1^a^	3.1 ± 1.1^a^	3.3 ± 1.2^a^	

^a^ Generalized Estimating Equation (GEE). *p*-value = 0.777, ^A^ Paired t-test. *p*-value = 0.641

Values are presented as mean ± SD. A *p*-value<0.05 indicates statistical significance

## Discussion

Despite the advancement in anesthetic techniques and perioperative care, aspiration remains an important complication in anesthesia. In fact, pulmonary aspiration was the third most common malpractice claim in the United States from 1990 to 2007 [32]. Point-of-care gastric ultrasound is emerging as a bedside tool that can provide both qualitative and quantitative information regarding a patient’s prandial status [9, 12–16].

Our study showed the diagnostic accuracy of qualitative gastric ultrasound assessment to be as high as 96% when performed by highly skilled regional anesthesiologists. Our finding supports the notion that bedside qualitative gastric ultrasound is relatively simple to master when the sonographer receives appropriate training [18, 33]. The percentage of correct diagnosis in our study (96%) is higher than the 79% reported by Arzola, *et al*. who also enrolled anesthesiologists [18]. One plausible explanation for this marked difference between studies might be differences in the level of ultrasound experience. Arzola, *et al*. enrolled anesthesiologists, including two anesthesia staff and four anesthesia fellows. In comparison, both anesthesiologists in our study are regional anesthesia specialists, which is a subspecialty that is highly proficient with the use of ultrasound.

The number of incorrect diagnoses in our study was too small to meaningfully compare with previously published studies. Both anesthesiologists correctly diagnosed all participants in the ‘fluid’ group, and one anesthesiologist misdiagnosed one volunteer in the ‘empty’ group, and the other anesthesiologist misdiagnosed one volunteer in the ‘solid’ group. Previous studies suggested the solid-containing antrum to be the most easily diagnosed due to its distinct ‘frosted-glass’ appearance [18, 33]. In the present study, one anesthesiologist misidentified an empty stomach as containing solid content. This might be explained by the presence of ‘frosted glass artifact’ due to baseline air in the gastric antrum [15, 34]. Another anesthesiologist reported a false-negative result by identifying a solid-containing antrum as empty. The aforementioned errors that occurred in our study demonstrate the inherent potential fallibility of subjective diagnostic testing. Kruisselbrink, *et al*. were the first to attempt to systematically examine the diagnostic accuracy of gastric ultrasound when performed by a single experienced gastric sonographer [34]. Given a pretest probability of 50%, the reported sensitivity and specificity were 1.0 and 0.975, respectively. Those authors recommended that gastric ultrasonography only be used in uncertain clinical scenarios [34]. Performing an ultrasound assessment without an indication might lead to unnecessary cancellation or postponement in the event of false-positive results, and a false-negative result could lead to a harmful act.

Regarding the qualitative assessment, a previous study by Arzola, *et al*. reported point-of-care gastric ultrasound to be a procedure of medium complexity [18]. In the present study, both anesthesiologists were able to achieve competence with a 90% success rate after only 9 scans as measured by a CUSUM analysis. This is in contrast to the study by Arzola, *et al*. whose sonographers needed 24 cases to achieve competence. As previously mentioned, this difference between studies may be due to differences in the level of ultrasound experience between the two groups of anesthesiologists. Our anesthesiologists are both regional anesthesiologists, and this anesthesia subspecialty is known to have a high level of proficiency in ultrasonography. The different distribution of volunteers between studies may have also contributed to this disparity. Another possible factor is that different types of gastric content may appear differently on ultrasound [18, 33]. Having acknowledged and described these variable factors, the much earlier achievement of competence observed in the present study should be interpreted with some thoughtful reservation.

Previous studies reported qualitative assessment of gastric content to be highly accurate, and that it can be easily learned by inexperienced gastric sonographers [18, 33]. However, other recent studies have shifted the focus towards quantifying fluid volumes to assess the risk of aspiration [12, 13, 15, 16, 34]. Although controversy still exists, it has been suggested that a gastric volume of <1.5 ml/kg is commonly found in fasted individuals, and that this content volume poses negligible aspiration risk [9]. Various mathematical models have been proposed to quantify the volume of gastric contents. The mathematical model used in our study demonstrated high reliability for predicting gastric volumes of up to 500 ml in non-pregnant individuals with a BMI <40 kg/m^2^ [13]. This model was developed using a ‘gold standard’ method of measuring gastric volumes, which is direct suction under gastroscope. Subsequent study adopted this mathematical model to measure gastric contents using both two-diameter method and free-tracing method, and they found near-perfect agreement for both intra- and interrater reliability (intraclass correlation coefficient > 0.8) [14]. Those authors concluded that quantification of gastric contents by ultrasound is highly reproducible; however, all three raters in that study had prior experience in gastric ultrasound. The amount of training required for a reliable quantitative assessment in novice gastric sonographers has yet to be determined. To our knowledge, this is the first study in interrater reliability conducted in anesthesiologists with no previous experience in gastric ultrasound.

Our study demonstrates a wide spectrum of correlation among different volumes of fluid, ranging from poor (ICC <0.5) to moderate (ICC 0.5–0.75) reliability [31]. Importantly – none of our quantitative assessment results are considered clinically acceptable. For the validated mathematical model to be reliable, the operator needs to strictly follow the standardized protocol. A still image has to be captured between peristaltic contractions of the stomach at the level of the aorta or the inferior vena cava. However, it was reported that obtaining an optimal image can be difficult to accomplish – even by an experienced sonographer [35]. Alternatively, a 3-point system for the grading of gastric volume was shown to have the ability to differentiate high and low volume states [13, 16]. Such stratification based on qualitative assessment might prove to be simpler whilst remaining clinically consequential. In the present study, the anesthesiologists had no difficulty identifying the relevant anatomical structures. The problem they encountered was selection of the most appropriate plane for volume calculation. Slight sliding/misdirection of the ultrasound probe could result in different static images that would lead to inaccurate volume calculation. Moreover, we observed that the size of the stomach also depends on the phase of respiration. Our finding highlights the well-established fact that ultrasound is operator-dependent. More training is required before novice gastric sonographers can accurately quantify gastric volume for aspiration risk stratification.

This study has several mentionable limitations. First, since the two evaluated anesthesiologists are both regional anesthesiologists, our results may not be representative of general anesthesiologists or other subspecialist anesthesiologists. However, it is arguably regional anesthesiologists who often manage patients with insecure airway [23]. Second, owing to time constraint and logistical issues, the gastric volume measurement was compared against the actual ingested volume. It has been proposed that the ‘gold standard’ of gastric volume measurement ought to be the volume of content suctioned under direct vision by gastroscope [13]. A comparison with the ingested volume fails to recognize the baseline gastric juice content, which is usually present even in adequately fasted patients [36, 37]. Moreover, gastric emptying might start immediately after the fluid has been ingested. The latter concern was addressed by substituting water with apple juice which empties less quickly. Despite these acknowledgements, the mean difference in volume for both anesthesiologists was still relatively substantial. Together with unsatisfactory interrater agreement, this study still strongly indicates that quantification of gastric content is a complex procedure that requires more training. Third and last, the fact that we enrolled healthy volunteers could limit the generalizability of our findings to unhealthy patient populations.

Fasting guidelines have remained a cornerstone of anesthesia practice even though knowledge exists that gastric emptying time is affected by various medical and surgical conditions [2, 3, 9–11], and that it varies among individuals [38]. An uncertain stomach condition could lead to an undesirable complication or unnecessary airway intervention. The noninvasive nature of ultrasound and its ability to provide rapid information suggests ultrasound as a promising and valuable imaging modality during the perioperative period. Gastric ultrasound is among the many expanding applications of PoCUS in anesthesia [39]. This increasing use of ultrasound in a variety of settings suggests the need for improved ultrasonography skills among anesthesiologists. Whether or not ultrasound will influence change in the current practice of aspiration risk assessment and stratification requires further study.

## Conclusion

The results of this study indicate that qualitative ultrasound assessment of gastric content is highly accurate and can be easily learned by highly competent regional anesthesiologists. This could potentially further the education of qualitative gastric ultrasound among trainees in our department. In contrast, quantification of gastric volume by novice anesthesiologist gastric sonographers is more complex and requires more training. Future research is needed to determine the optimal protocol for training sonographers in quantitative gastric ultrasound assessment. In the meantime, the authors propose that ultrasound-based gastric volume measurement as measured by an inadequately trained gastric sonographer should not override the published fasting guidelines when making a clinical decision.

## Data Availability

The datasets used and/or analysed during the current study are available from the corresponding author on reasonable request.

## Abbreviations

PoCUS: point-of-care ultrasound
CUSUM: Cumulative sum
ICC: Intraclass correlation coefficient
CSA: cross-sectional area
